# Rhynchophylline Alleviates Hyperactivity and Cognitive Flexibility Impairment Associated With Inhibition of Inflammatory Responses in Mice That Partly Lack the Dopamine Transporter Protein

**DOI:** 10.1002/brb3.70121

**Published:** 2024-11-11

**Authors:** Jijun Li, Bojun Chen, Zai‐wang Li, Yi Wang, Ian Alberts, Kexing Sun, Xiaohong Li

**Affiliations:** ^1^ Department of Integrative Medicine on Pediatrics, Shanghai Children's Medical Center Shanghai Jiao Tong University School of Medicine Shanghai P.R. China; ^2^ Guizhou Branch of Shanghai Children's Medical Center Guizhou Provincial People's Hospital Guiyang P.R. China; ^3^ The Second Clinical Medical School Yunnan University of Chinese Medicine Kunming Yunnan P.R. China; ^4^ Department of Neurology, Shenzhen People's Hospital The Second Clinical Medical College of Jinan University The First Affiliated Hospital of Southern University of Science and Technology Shenzhen P.R. China; ^5^ Department of Otolaryngology Yunnan University of Traditional Chinese Medicine Kunming P.R. China; ^6^ Department of Natural Sciences, LaGuardia CC City University of New York New York New York USA; ^7^ Department of Neurochemistry New York State Institute for Basic Research in Developmental Disabilities New York New York USA

**Keywords:** DAT− mice, dopamine transporter, hyperactivity and cognitive flexibility impairment, inflammatory, rhynchophyllin

## Abstract

**Background and aims:**

Rhynchophylline (RHY) can alleviate some cognitive flexibility impairment and stereotyped behavior for attention‐deficit hyperactivity disorder (ADHD) and Tourette syndrome (TS) patients as one of a key extract and an active ingredient in Ningdong granule (NDG), which is a Traditional Chinese medicine (TCM) preparation widely used in the treatment of ADHD and TS children in China; however, the underlying mechanism is not well understood. Therefore, this study aimed to evaluate how RHY alleviates hyperactivity and cognitive flexibility impairment while inhibiting inflammatory responses in mice that partly lack dopamine transporter protein (DAT− mice).

**Methods:**

Male DAT− mice were randomly divided into the RHY group (*n* = 8) and administered RHY (30 mg/kg) in the DAT− group (*n* = 8) and administered saline (i.p., 10 mL/kg) in wild‐type (WT) mice as the WT control group (*n* = 8). Hyperactivity and cognitive flexibility impairment were evaluated by the open field test (OFT) and the Morris water maze (MWM) test. The levels of the inflammatory factors of tumor necrosis factor‐α (TNF‐α) and interleukin‐1β (IL‐1β) in cortical homogenates were tested by enzyme‐linked immunosorbent assays (ELISA) after 8 weeks of treatment with RHY. In vitro, primary microglia and astrocytes extracted from the cortices of DAT− neonatal mice and WT neonatal mice were treated with lipopolysaccharide (LPS) (1 mg/mL) to induce neuroinflammatory responses and with RHY (20 mM) for 48 h. The levels of the inflammatory factors TNF‐α, IL‐1β, inducible nitric oxide synthase (iNOS), and cyclooxygenase‐2 (COX2) in the culture medium were measured at 6 h, 24 h, and 48 h after treatment with LPS and RHY.

**Results:**

RHY ameliorated hyperactivity and cognitive flexibility impairment in DAT− mice and inhibited the expression of the inflammatory factors TNF‐α, IL‐1β, iNOS, and COX‐2 in microglia and astrocytes in vitro, and also inhibited the expression of TNF‐α and IL‐1β in cortical homogenates after 8 weeks of treatment.

**Conclusion:**

RHY improved hyperactivity and cognitive flexibility impairment through inhibiting inflammatory responses in DAT− mice.

## Graphical abstract

(1) RHY reduced hyperactivity and improved cognitive flexibility in DAT‐deficient mice. (2) RHY inhibited inflammatory factor expression in vivo (TNF‐α, IL‐1β) and in vitro (TNF‐α, IL‐1β, iNOS, COX‐2). (3) This study suggests RHY's therapeutic potential for neuropsychiatric disorders involving dopamine transporter deficiency through anti‐inflammatory effects.

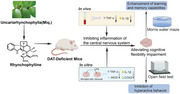

## 1 Background

Hyperactivity and cognitive flexibility impairment, which often manifest as impulsivity, sustained attention impairment, and social‐behavioral difficulties, are the core abnormal behaviors observed in children with attention‐deficit hyperactivity disorder (ADHD) (Wylock et al. 2023; Tripp and Wickens 2009; Lambez et al. 2020). The biological mechanism and etiology of ADHD are not fully understood. Previous studies have shown that disturbance of dopamine (DA) metabolism is involved in the pathology of ADHD (Del Campo et al. 2011; Dresler et al. 2014; Adinolfi et al. 2018; Cannon Homaei et al. 2022). Changes in DA‐mediated behaviors, psychostimulant effects, and impairment of DA activity are associated with neurological disorders (Adriani et al. 2012). Studies on the neurobiological basis of ADHD using mouse and rat models lacking the dopamine transporter protein (DAT) (DAT− mice/rats) and DAT gene knockout (KO) animals have shown that DAT plays a key role in DA metabolism and signaling (Zhuang et al. 2001; Russell 2007; Deng et al. 2015). Moreover, early neuroinflammatory responses in the central nervous system (CNS) are strongly correlated with some neurodevelopmental diseases, such as ADHD (Song et al. 2021). Environmental effects are generally nonspecific, as various psychiatric outcomes are associated with these factors; in many instances, environmental risk factors lead to ADHD accompanied by other psychiatric symptoms and conditions (Dunn, Nigg, and Sullivan 2019; Song et al. 2020; Han et al. 2021).

Currently, the psychostimulant drug methylphenidate (MPH), which blocks the reuptake of DA and norepinephrine into presynaptic neurons and subsequently increases, the levels of extraneuronal DA, is one of the most commonly used and effective agents for alleviating the main symptoms of ADHD (Pringsheim et al. 2015; Stuckelman et al. 2017; Faraone. 2018; Jaeschke, Sujkowska, and Sowa‐Kućma 2021). Our research over several years has proven that the traditional Chinese medicine (TCM) preparation Ningdong granule (NDG) can relieve hyperexcitability in patients with ADHD and Tourette syndrome (TS) (A. Y. Li et al. 2009; J. Li et al. 2020, J. J. Li 2011; Lv et al. 2009; L. Zhao et al. 2020).

**FIGURE 1:**
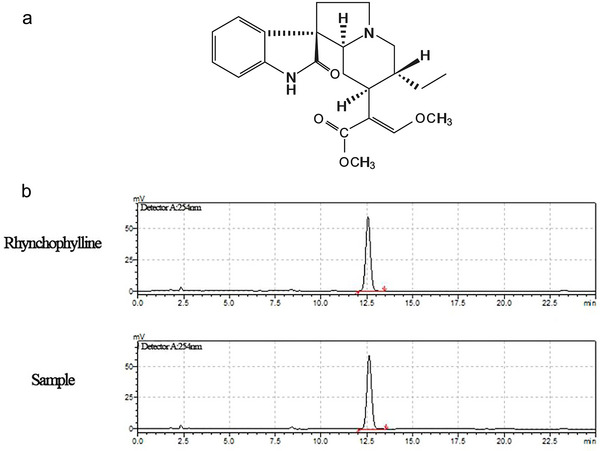
(a) Chemical structure of RHY. (b) HPLC of the standard substance of RHY (Sigma, Japan) and RHY of the current experimental sample (Zelang Biotechnology, Nanjing, China).

As a key active component of NDG, rhynchophylline (RHY) is an important active tetracyclic oxindole alkaloid that is isolated from a Chinese herb *Uncaria rhynchophylla* (Miq.) and is commonly used as a TCM herbal product to treat CNS disorders such as epilepsy, hypertension, ADHD, and other neurodevelopmental diseases (Yang et al. 2020). RHY has been proven to improve cognitive impairment in Alzheimer's disease (AD) model in rats (Shao et al. 2015; Yang et al. 2018) and learning and memory impairments induced by D‐galactose in AD model mice (Xian et al. 2014). RHY has also exerted its effects against these diseases by inhibiting inflammation for CNS protection (He et al. 2014; Song et al. 2012; Zhou and Zhou 2010; Huang et al. 2014; Jiang et al. 2021), conferring neuroprotection and increasing neurotrophin levels in the CNS, in which the mechanisms may involve activating the PI3K/Akt signaling pathway (Zheng et al. 2021). The chemical structure and high‐performance liquid chromatography (HPLC) chromatogram of RHY are shown in Figure 1a,b, respectively. However, it is not known whether there is a link for RHY between the improving hyperactivity and inhibiting some of inflammatory cytokines. Therefore, the main aim of this study was to evaluate the possible mechanism by which RHY relieves hyperactivity and cognitive flexibility impairment and inhibits inflammatory responses in DAT− mice.

## 2 Methods

### 2.1 Animals’ Ethics

The mice were maintained and handled according to the guidelines of the Animal Ethical Committee of Shanghai Children's Medical Center, Shanghai Jiao Tong University School of Medicine (No. 20170721B), and adequate measures were taken to minimize animal discomfort.

### 2.2 Animal Administration

**FIGURE 2:**
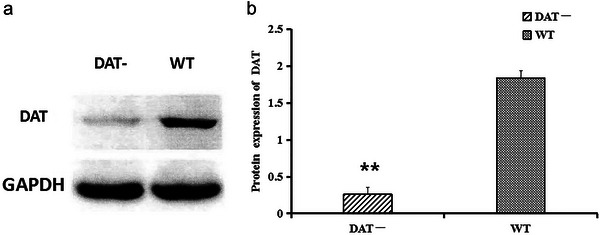
DAT protein expression measured by western blotting for DAT− mice versus WT mice. The DAT mRNA band is shown in (a), and relative expression levels are shown in (b). **p < 0.01.

The homozygote of DAT knockdown (DAT−) mice was a generous gift from the laboratory of Dr. Shining Deng of the Developmental and Behavioral Pediatric & Child Primary Care Department, Xinhua Hospital, Shanghai Jiao Tong University School of Medicine. The DAT protein expression in striatal tissue was measured by western blot (Zhuang et al. 2001; Deng et al. 2015). Sixteen male DAT− mice were randomly divided into the DAT− (DAT−, *n* = 8) and RHY + DAT− (RHY, *n* = 8) groups and housed in an air‐conditioned animal room on a 12‐h light/dark cycle at a temperature of 22 ± 2°C and a humidity of 50 ± 10%. Eight WT mice were used as healthy controls (Deng et al. 2015). Furthermore, eight female DAT− and WT mice were used for breeding to obtain mice for in vitro experiments. The mice were provided free access to food and tap water and were maintained for 1 week before the start of the experiment. The protein level of DAT in the cortices of DAT− mice was about 20% of that in the cortices of wild‐type (WT) mice (Figure 2a,b).

RHY (Nanjing Zelang Biology, China) prepared in saline was administered daily at a dose of 30 mg/kg by intraperitoneal (i.p.) injection (10 mL/kg) to mice in the RHY + DAT− group (Huang et al. 2014; Xian et al. 2017), and mice in the DAT− group were administered the same volume of saline. Healthy WT mice in the WT group were maintained under normal conditions with no drug treatment. All the treatment for mice was done between 8 and 10 a.m. every day for 8 weeks.

Behavioral tests were performed before injection (Week 1) and then once a week after treatment. After 8 weeks of drug administration, serum was isolated from the caudal vein, and the amount of TNF‐α, IL‐1β, iNOS, and COX‐2 in the serum was measured using commercially available enzyme‐linked immunosorbent assay (ELISA) kits. Finally, all the mice were euthanized under anesthesia using the mixture of ketamine (50 mg/kg) and xlazine (15 mg/kg) by intraperitoneal injection, and the cortices of the mice were quickly collected and placed in 2 mL of ice‐cold sucrose buffer (0.32 M sucrose in 5 mM HEPES, pH 7.4). The mouse tissues were homogenized by using a Teflon glass homogenizer, and the homogenized samples were centrifuged at 1000 × *g* for 10 min at 4°C. After centrifugation, the supernatant liquid was collected and centrifuged again at 12,000 × *g* for 20 min, and the subsequent supernatant was used for measurement of TNF‐α and IL‐1β levels by ELISA. Finally, all the remaining brain tissues were stored at −80°C.

### 2.3 Animals' Behavioral Tests

#### 2.3.1 Open Field Test (OFT)

The OFT was performed to evaluate whether the mice treated with RHY exhibited changes in spontaneous locomotor activity and hyperactivity. The OFT was performed according to previously reported protocols (Deng et al. 2015; Cui et al. 2004; W. X. Zhao et al. 2017). The test was conducted in a square Plexiglas apparatus (40 × 40 × 35 cm) under diffuse light. A digital camera was mounted directly above the apparatus, and images were captured at a rate of 5 Hz. The data were quantified using the Ethovision video tracking system (Noldus Information Technology, Wageningen, Netherlands). Each mouse was gently placed in the apparatus and allowed to explore freely for 15 min. The spontaneous locomotor activity of the mice in the OFT was assessed by recording their trajectories and measuring the distance traveled, the movement duration, the speed, and the number of crossings. After each trial, the apparatus was cleaned, and the animal was returned to its home cage.

#### 2.3.2 Morris Water Maze (MWM) Test

The MWM test, which is used to assess spatial learning, spatial memory, and cognitive flexibility, was performed as described previously with minor modifications (Morris et al. 1982; Vorhees and Williams 2006; Chen et al. 2015). All the experimental mice were tested once a day during the treatment. The apparatus was placed in a room with several visual cues to allow the mice to orient themselves within the water maze, and the maze was divided into four quadrants. The apparatus consisted of a circular swimming pool (100 cm in diameter, 50 cm high) with black ABS engineering plastic walls and floors (Shanghai XinRuan Biotechnology Co., Ltd.). The swimming pool was filled with water maintained at a temperature of 24°C–26°C to a depth of 30 cm, and the experimental conditions (room extra cues, water temperature, room temperature, light) were kept constant throughout the experiment. A different shape was placed in each quadrant (1.5 cm below the water level) as a visual cue to help the mice find the escape platform. The place navigation and spatial probe phases of the MWM test were performed successively.

The escape platform was kept in a fixed position throughout the place navigation phase. The platform was the only method of escape from the water, it was hidden underwater, so the mice had to search for it. Place navigation trials were performed three times per day for 5 consecutive days, and the probe trial was performed on the 6th day. In the navigation trials, the mice were released into the water facing the pool wall in randomly selected starting positions. The mice were allowed to swim for a maximum of 120 s until they found the platform. If a mouse failed to find the platform within 120 s, it was gently placed on the platform and allowed to stay on it for 30 s before the next trial. On the day of the probe trial, the platform was removed, and each mouse was released into the pool from the same position. The swimming paths of the mice were recorded for 60 s by a video camera mounted on the ceiling. The video data were analyzed by the VisuTrack software package (Xin Ruan, Shanghai, China).

### 2.4 Animals’ Cytological Experiments

#### 2.4.1 Primary Mouse Mixed Glial Cell (Astrocyte and Microglia) Culture and Treatment

Primary mouse mixed glial cells (astrocytes and microglia) were prepared from the cortices of neonatal DAT− mice (*n* = 4) and neonatal WT mice (*n* = 4) on postnatal Day 1. In brief, the brain tissue samples were triturated into single cells in a cell culture medium supplemented with 10% fetal bovine serum (FBS) and antibiotics (100 U/mL penicillin and 100 µg/mL streptomycin) at 37°C for 3 days.

The cell culture medium was changed 2 days after plating and every 3 days thereafter until the microglia and astrocytes reached confluence. Then, the microglia and astrocytes were plated in 96‐well plates. DAT− mouse cells in the LPS group (*n* = 4) were treated with lipopolysaccharide (LPS) (1 mg/mL), DAT− mouse cells in the RHY group (*n* = 4) were treated with LPS (1 mg/mL) and RHY (20 mM) for 48 h, and the DAT− mouse cells in the DAT− group (*n* = 4) were left untreated. Untreated primary microglia and astrocytes from 4 neonatal WT mice (*n* = 4) were used as the WT group in the in vitro experiment.

#### 2.4.2 Immunohistochemistry

After LPS or RHY treatment, the astrocytes and microglia remaining in the culture medium were identified by immunostaining with anti‐glial fibrillary acidic protein (GFAP) and anti‐CD11b antibodies (Santa Cruz, Shanghai). After incubation with the primary antibodies at room temperature for 6 h, the cells were incubated with a fluorescein isothiocyanate (FITC, green)‐ or Cyanine 3 (Cy3, red)‐conjugated secondary antibody (Santa Cruz, Shanghai) for 2 h. Then, the cells were stained with 4*′*,6‐diamidino‐2‐phenylindole (DAPI) (blue) for 3 min and observed using an inverted fluorescence microscope (Leica DMI6000B, Germany).

#### 2.4.3 Analysis of Inflammatory Factors by ELISA

Mouse cortical tissue was collected and homogenized, and the supernatant was collected after centrifugation for measurement of TNF‐α and IL‐1β levels by ELISA. Mouse cortical tissue was homogenized in RIPA buffer supplemented with a protease and phosphatase inhibitor cocktail (Applygen, Beijing, China) on ice. The protein concentrations in the supernatants were determined after centrifugation at 20,000 rpm for 20 min with a BCA Protein Assay Reagent Kit (Thermo, USA). A total of 5 µL of protein from each sample was used for analysis.

The levels of the inflammatory factors TNF‐α, IL‐1β, iNOS, and COX‐2 in the culture medium were measured at 6, 24, and 48 h after treatment with LPS or RHY using ELISA kits according to the manufacturer's instructions. Briefly, antigen standards and samples were added to the wells of 96‐well plates precoated with primary antibodies.

After adding Biotin Conjugate Reagent and Enzyme Conjugate Reagent to each well, the plates were incubated at 37°C for 60 min and then rinsed three times with distilled water. After the chromogenic reaction, the absorbance was measured at 450 nm using a microtiter plate reader (Thermo Fisher, Shanghai) within 30 min.

### 2.5 Statistical Analysis

All data are presented in the text as the means ± the standard deviations. All analyses were performed using the SPSS statistical software package (version 23.0, PSS Inc., Chicago, IL, USA), and *p* < 0.05 was considered statistically significant. The Shapiro‒Wilk test was used to verify the normality of the data, and Levene's test was utilized to analyze the homogeneity of variance. Data exhibiting a normal distribution were statistically analyzed by one‐way analysis of variance (ANOVA). All other data were analyzed by the K independent samples test. If the data showed homogeneity of variance, differences among multiple groups were evaluated statistically by ANOVA. Multiple comparisons were made by the least significant difference (LSD) method if the data exhibited variance homogeneity, otherwise, they were made by the Games‐Howell method. The correlation analysis was conducted using Pearson correlation coefficients to examine the relationship between the decreased levels of inflammatory factors and the enhanced behavioral outcomes.

## 3 RESULTS

### 3.1 Assessment of Mouse Behaviors

#### 3.1.1 OFT

**FIGURE 3:**
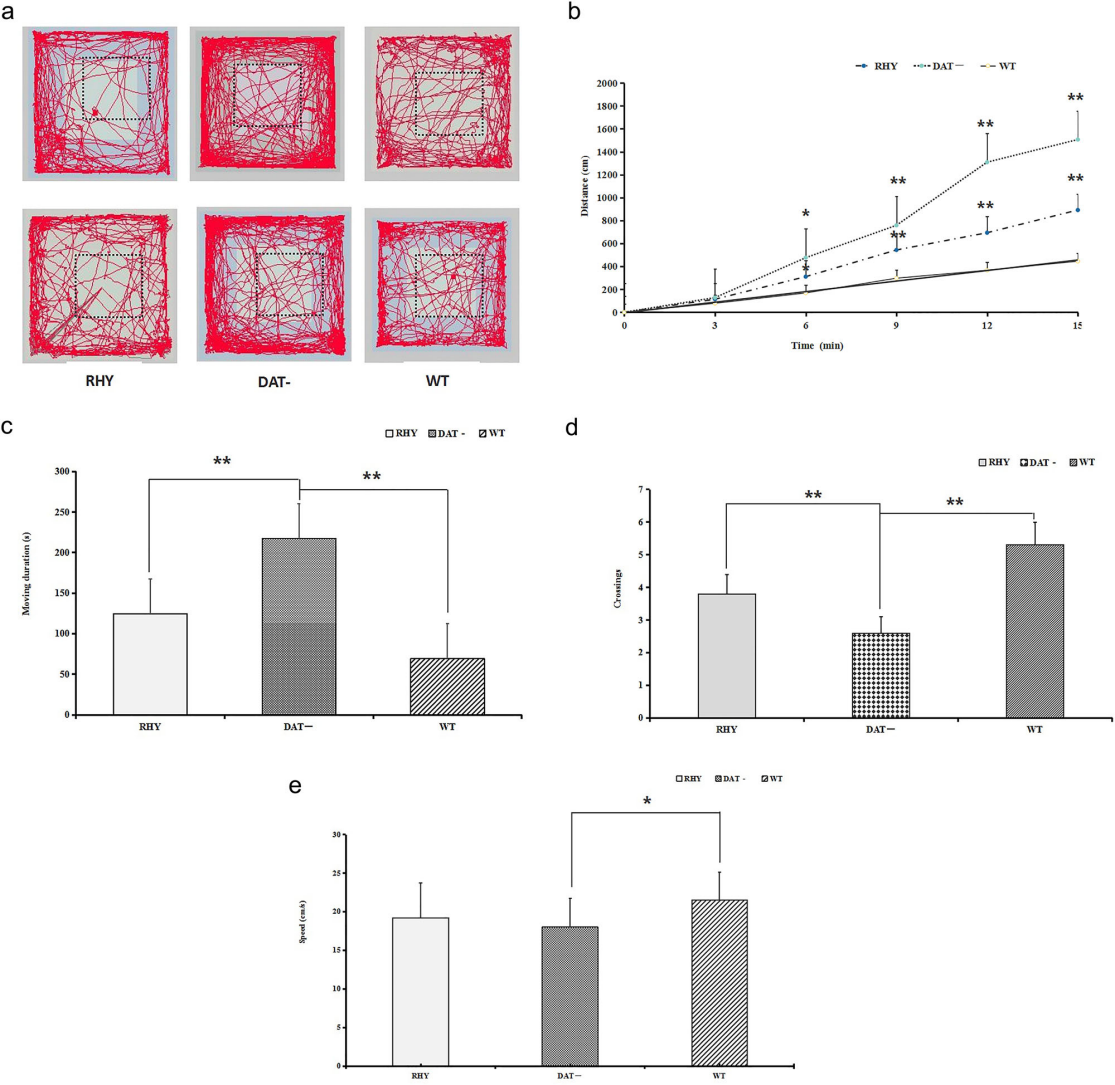
Spontaneous locomotor activity of RHY, DAT, and WT mice in the OFT. The results for the LPS group: (a) distance traveled; (b) movement duration; (c) speed; (d) and (e) number of crossings; **p < 0.01.

To assess whether the changes in behavior after RHY administration were attributable to changes in spontaneous locomotor activity, the OFT was conducted. Mice in the DAT− group traveled a much greater distance (Figure 3a,b) and moved for a longer amount of time (**p < 0.01, Figure 3c) with fewer crossings (**p < 0.01, Figure 3d) within 15 min than those in the RHY and WT groups. However, lower moving speed in DAT− mice than that in WT mice (*p < 0.05), and no difference in DAT− mice and RHY mice (Figure 3e). The results suggest that RHY could alleviate hyperactivity, as it reduced the distance traveled and the duration of movement, but no change shown in the average velocity of motion.

#### 3.1.2 MWM Test

**FIGURE 4:**
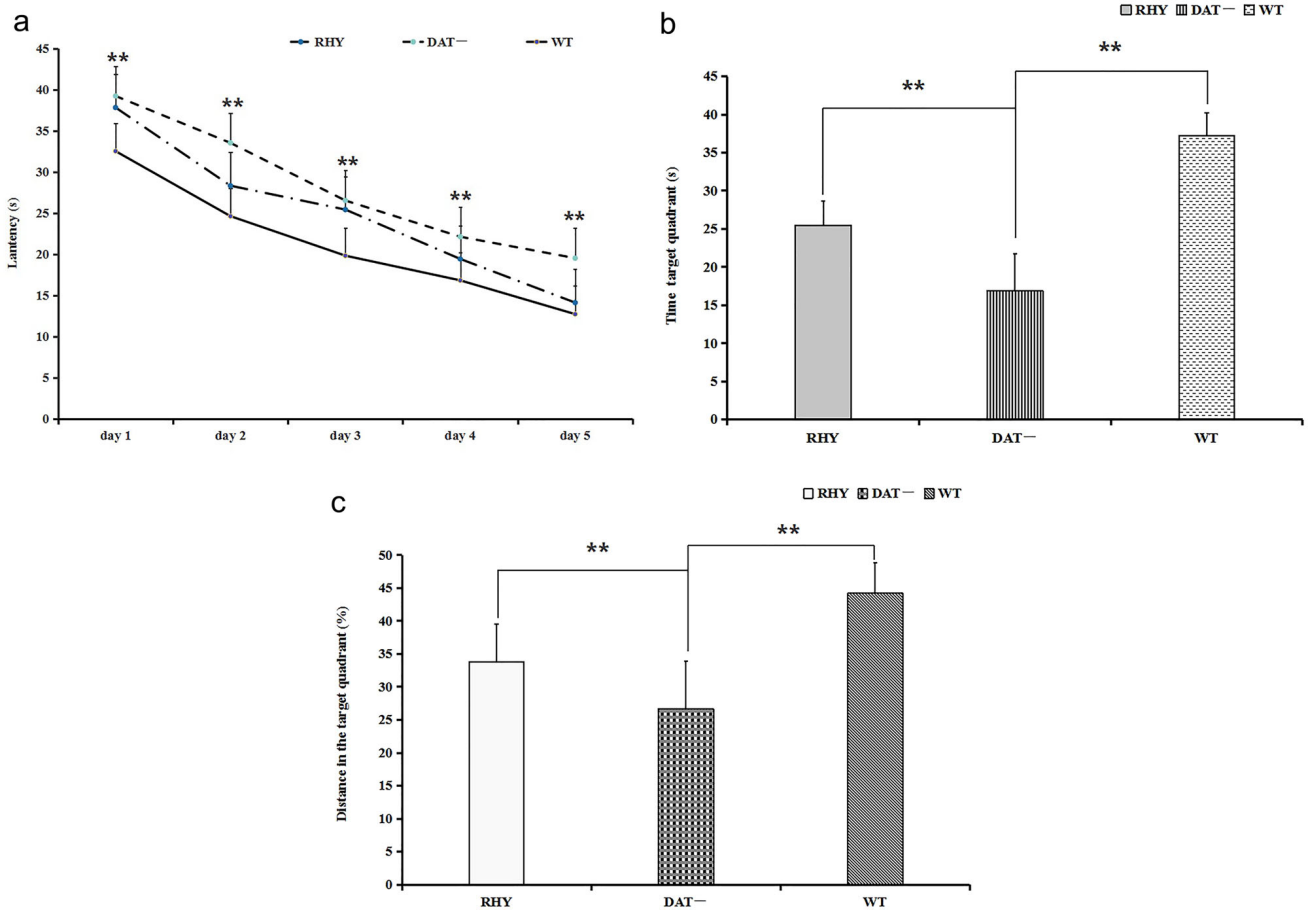
Spatial learning ability and memory in the MWM test: (a) Learning phase: latency to find the platform (**p < 0.01), (b) Probe test: time spent in the target quadrant, and (c) Probe test: distance traveled in the target quadrant, **p < 0.01.

The cognitive flexibility of the experimental mice, i.e., their spatial learning ability and memory, including their ability to collect, process, sort, memorize, and integrate visual information, were assessed using the MWM test. As shown in Figure 4a, the escape latency of each of the three groups decreased significantly from Days 1 to 5 (days 1–5 in the DAT− group: 39.2 ± 7.5, 33.5 ± 5.6, 26.5 ± 4.3, 22.1 ± 3.7, and 19.5 ± 4.1 s; Days 1 to 5 in the RHY‐ group: 37.8 ± 4.8, 28.3 ± 5.1, 25.4 ± 3.6, 19.4 ± 3.8, and 14.1 ± 3.2 s; Days 1 to 5 in the WT group: 32.5 ± 2.7, 24.6 ± 1.9, 19.8 ± 3.2, 16.8 ± 3.8, and 12.7 ± 3.3 s). During the learning phase, the latency to find the platform was significantly longer in the DAT− group than in the RHY and WT groups (**p < 0.01) (Figure 4a). In the probe test, the mice in the RHY and WT groups spent a longer amount of time (Figure 4b) and traveled a longer distance in the target quadrant (Figure 4c) than the DAT− mice (**p < 0.01).

### 3.2 Cytological Experiments

#### 3.2.1 LPS‐Treated Microglia and Astrocytes

**FIGURE 5:**
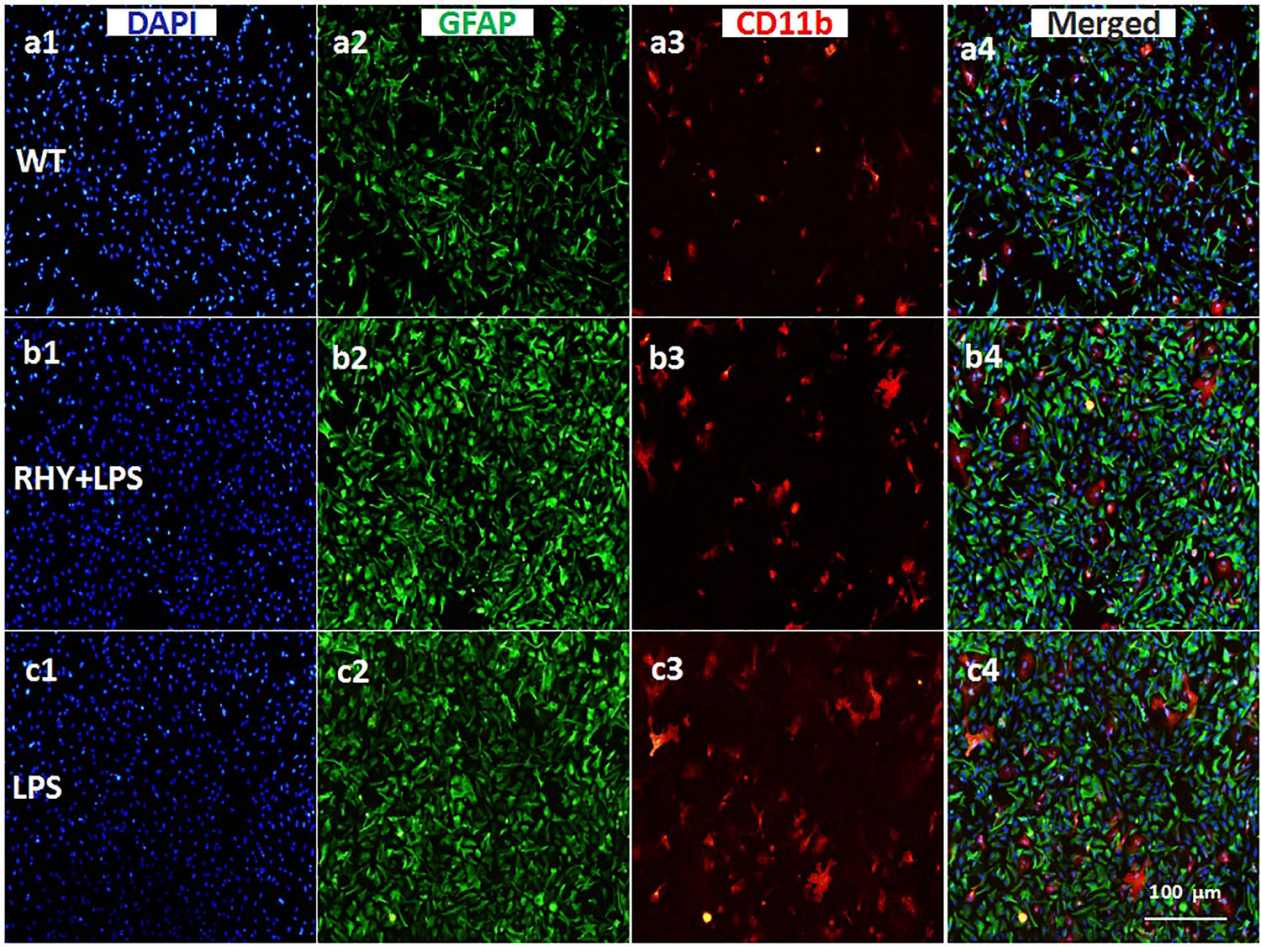
Astrocytes and microglia remaining in the cell culture medium for the RHY + LPS, LPS, and WT groups: DAPI staining (a1–c1), GFAP immunostaining (a2–c2), CD11b staining (a3–c3), merged images (a4–c4), and the scale bar = 100 µm.

As shown in Figure 5, cultured primary astrocytes and microglia were subjected to immunofluorescence staining for GFAP and CD11b in vitro at 6, 24, and 48 h. The density of microglia and astrocytes was lower in the LPS group than in the RHY‐treated and WT groups. Weaker GFAP fluorescence and a lower density of GFAP‐positive cells were also observed in the LPS group than in the other two groups (Figure 5a2,b2,c2).

#### 3.2.2 Measurement of TNF‐α and IL‐1β Levels in Cortical Tissue Homogenates

**FIGURE 6:**
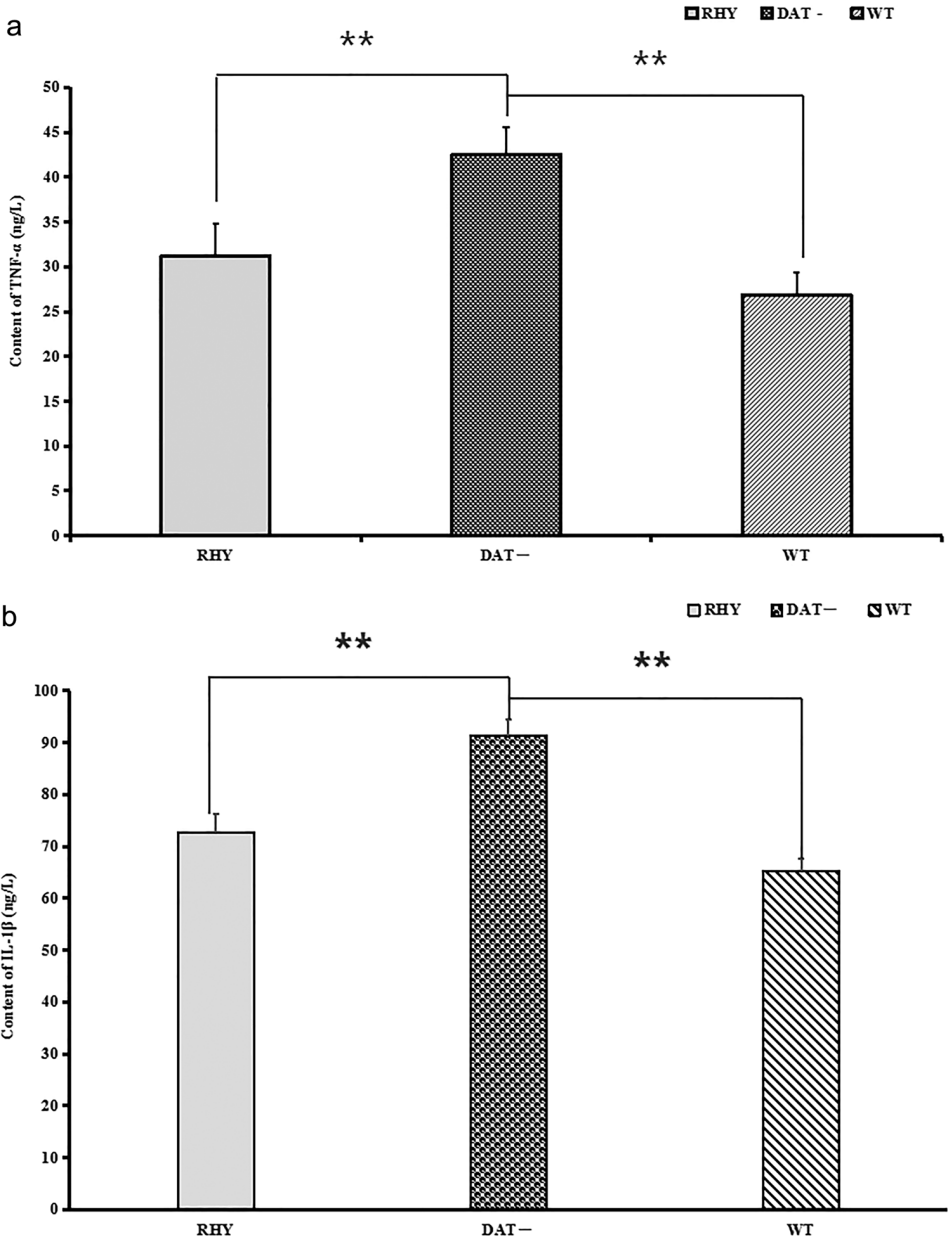
Content of TNF‐α and IL‐1β in cortical homogenates from the mouse groups: (a) TNF‐α and (b) IL‐1β; **p < 0.01.

As shown in Figure 6a,b, the protein expression levels of TNF‐α and IL‐1β in cortical tissue homogenates were decreased after the 8‐week treatment period in the RHY group (TNF‐α: 31.2 ± 3.6 ng/L; IL‐1β: 72.6 ± 11.3 ng/L) compared to the DAT− group (TNF‐α: 42.5 ± 3.1 ng/L; IL‐1β: 91.3 ± 13.8) (**p < 0.01). There was no significant difference in TNF‐α and IL‐1β protein levels between the RHY group and the WT group (TNF‐α: 31.2 ± 3.6 ng/L; IL‐1β: 65.2 ± 9.6 ng/L) (**p < 0.01).

#### 3.2.3 Levels of TNF‐α, IL‐1β, iNOS, and COX‐2 in the Culture Medium

**FIGURE 7:**
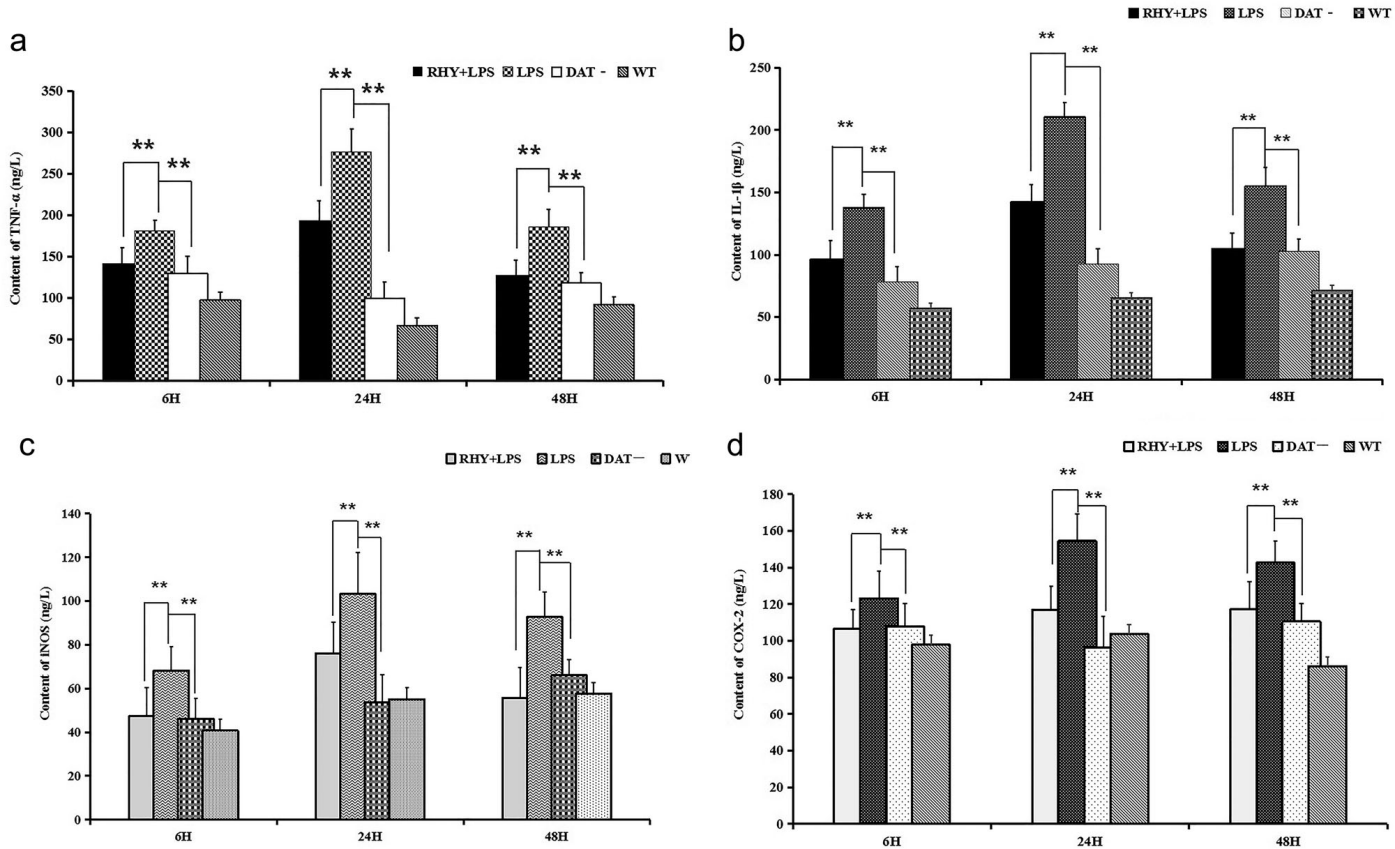
Content of TNF‐α, IL‐1β, iNOS, and COX‐2 in the serum: (a) TNF‐α; (b) IL‐1β; (c) iNOS; (d) COX‐2; **p < 0.01.

The concentration histograms of the inflammatory factors showed a clear decreasing tendency after 48 h of treatment with RHY. As shown in Figure 7a, the concentration of TNF‐α was lower in the RHY group (6 h: 141.5 ± 21.3; 24 h: 193.6 ± 12.7; 48 h: 127.9 ± 19.6) than in the LPS group (6 h: 181.0 ± 19.9; 24 h: 276.5 ± 27.7; 48 h: 185.8 ± 23.3) at 6, 24, and 48 h (**p < 0.01); however, there was no significant difference in the TNF‐α concentration between the RHY group and the WT group (6 h: 97.4 ± 23.1; 24 h: 66.5 ± 21.9; 48 h: 91.6 ± 16.3) or the DAT− group (6 h: 129.2 ± 12.8; 24 h: 99.4 ± 21.0; 48 h: 117.7 ± 17.3).

Similarly, Figure 7b shows that the IL‐1β content in the culture medium of microglia and astrocytes from the RHY group (6 h: 96.2 ± 11.9; 24 h: 142.7 ± 10.6; 48 h: 104.8 ± 15.0) at 6, 12, and 24 h (**p < 0.01) was lower than that in the culture medium from the LPS group (6 h: 137.9 ± 12.3; 24 h: 210.5 ± 11.8; 48 h: 155.2 ± 13.5), but there was no significant difference in IL‐1β levels in the culture medium among the WT (6 h: 57.2 ± 12.8; 24 h: 66.5 ± 11.3; 48 h: 71.6 ± 14.2), DAT (6 h: 78.5 ± 9.8; 24 h: 92.8 ± 14.7; 48 h: 103 ± 12.4) and RHY groups

As depicted in Figure 7c the protein expression of the inflammatory factor iNOS in the culture medium of microglia and astrocytes from the RHY group (6 h: 47.3 ± 9.3; 24 h: 76.0 ± 10.9; 48 h: 55.8 ± 13.2) at 6, 12, and 24 h (*p < 0.05) was lower than that in the culture medium from the LPS group (6 h: 68.1 ± 12.4; 24 h: 103.2 ± 18.9; 48 h: 92.7 ± 14.2) (**p < 0.01); however, there was no significant difference in iNOS protein expression among the WT (6 h: 40.7 ± 9.4; 24 h: 55.1 ± 11.8; 48 h: 57.6 ± 13), DAT− (6 h: 46.2 ± 7.2; 24 h: 53.8 ± 11.3; 48 h: 66.1 ± 13.8) and RHY groups (Table 1).

Finally, Figure 7d illustrates that the concentration of COX‐2 in the RHY group (6 h: 106.7 ± 12.5; 24 h: 106.7 ± 12.5; 48 h: 117.2 ± 10.2) at 6, 12, and 24 h (**p < 0.01) was lower than that in the LPS group (6 h: 123.0 ± 17.1; 24 h: 154.5 ± 14.8; 48 h: 142.6 ± 13.0), and, again, there was no significant difference in the COX‐2 concentration among the WT group (6 h: 97.8 ± 12.4; 24 h: 103.7 ± 10.7; 86.1 ± 14.6), the DAT− group (6 h: 107.7 ± 9.8; 24 h: 96.2 ± 11.9; 48 h: 110.5 ± 15.2), and the RHY group (Table 1).

**TABLE 1:** Levels of TNF‐α, IL‐1β, iNOS, and COX‐2 in the culture medium for 48 h treatment (ng/L).

		RHY + LPS (*n* = 4)	DAT− (*n* = 4)	WT(*n* = 4)	LPS(*n* = 4)	*F*	*p*
TNF‐α	6 h	141.5 ± 21.3;	129.2 ± 12.8	97.4 ± 23.1	181.0 ± 19.9	12.358	** *p* < 0.01
	24 h	193.6 ± 12.7	99.4 ± 21.0	66.5 ± 21.9	276.5 ± 27.7	78.194	** *p* < 0.001
	48 h	127.9 ± 19.6	117.7 ± 17.3	91.6 ± 16.3	185.8 ± 23.3	16.949	** *p *< 0.001
IL‐1β	6 h	96.2 ± 11.9	78.5 ± 9.8	57.2 ± 12.8	137.9 ± 12.3	33.932	** *p *< 0.001
	24 h	142.7 ± 10.6	92.8 ± 14.7	66.5 ± 11.3	210.5 ± 11.8	107.885	** *p *< 0.001
	48 h	104.8 ± 15.0	103 ± 12.4	71.6 ± 14.2	155.2 ± 13.5	25.080	** *p *< 0.001
iNOS	6 h	47.3 ± 9.3	46.2 ± 7.2	40.7 ± 9.4	68.1 ± 12.4	6.091	** *p *< 0.001
	24 h	76.0 ± 10.9	53.8 ± 11.3	55.1 ± 11.8	103.2 ± 18.9	11.531	** *p* < 0.001
	48 h	55.8 ± 13.2	66.1 ± 13.8	57.6 ± 13	92.7 ± 14.2	6.315	* *p *< 0.05
COX‐2	6 h	106.7 ± 12.5	107.7 ± 9.8	97.8 ± 12.4	123.0 ± 17.1	2.507	0.109
	24 h	115.7 ± 11.3	96.2 ± 11.9	103.7 ± 10.7	154.5 ± 14.8	17.839	** *p* < 0.001
	48 h	117.2 ± 10.2	110.5 ± 15.2	86.1 ± 14.6	142.6 ± 13.0	12.037	** *p *< 0.001

*Note*: Comparison of TNF‐α, IL‐1β, iNOS, and COX‐2 levels in the culture medium among RHY + LPS, DAT−, WT, and LPS showed significant differences.

* *p* < 0.05, **p < 0.01.

#### 3.2.4 The Correlation Analysis of the Decreased Levels of Inflammatory Factors and the Enhanced Behavioral Outcomes in OFT and MWM Tests

The correlation analysis was conducted using Pearson correlation coefficients to examine the relationship between the decreased levels of inflammatory factors and the enhanced behavioral outcomes in OFT and MWM tests after RHY treatment, and the results are shown in Table 2.

**TABLE 2:** The correlation analysis between decreased inflammatory factors and improved behavioral outcomes after RHY treatment in OFT and MWM tests.

		OFT				MWM		
Decreased content of inflammatory factors		Traveled distance at 15 min (cm)	Moved time (s)	Crossings	Moving speed	Latency	Time spent	Distance in the target quadrant (%)
TNF‐α	13.6 ± 2.5	553.9 ± 54.5**	75.8 ± 16.0**	1.7 ± 0.5**	7.3 ± 1.6**	18.7 ± 3.5**	18.5 ± 6.6*	8.4 ± 2.7*
IL‐1β	8.6 ± 2.6	553.9 ± 54.5**	75.8 ± 16.0**	1.7 ± 0.5*	7.3 ± 1.6*	18.7 ± 3.5*	18.5 ± 6.6*	8.4 ± 2.7*
iNOS	8.5 ± 1.4	553.9 ± 54.5	75.8 ± 16.0	1.7 ± 0.5*	7.3 ± 1.6*	18.7 ± 3.5	18.5 ± 6.6*	8.4 ± 2.7*
COX‐2	10.5 ± 3.4	553.9 ± 54.5*	75.8 ± 16.0*	1.7 ± 0.5	7.3 ± 1.6*	18.7 ± 3.5	18.5 ± 6.6	8.4 ± 2.7

*Note*: The correlation analysis was calculated using Pearson correlation coefficients between decreased levels of inflammatory factors and enhanced behavioral outcomes in OFT and MWM tests.

* *p* < 0.05, **p < 0.01.

## 4 Discussion

Hyperactivity, impulsion, and cognitive flexibility impairment, including attention deficits and learning difficulties, are the core symptoms of ADHD; however, the pathology of ADHD remains largely unknown. Nevertheless, in this context, there is some emerging evidence regarding the pathophysiological changes associated with ADHD, including disturbance of DA metabolism (Rastedt, Vaughan, and Foster 2017; Reith et al. 2022), chronic neuroinflammation, and immune responses in the CNS (Reith et al., 2019; Han et al. 2021). DAT is an important monoamine transporter that plays a key role in the metabolism of DA and the regulation of neurotransmission by removing extracellular DA and modulating its reuptake into neurons to determine the duration of the effects of DA in local neural circuits (Rastedt, Vaughan, and Foster 2017; Mazei‐Robinson and Blakely 2006; Genro et al. 2010; Reith et al. 2022). The use of several rodent models, such as spontaneously hypertensive rats and mice lacking or partly lacking DAT (Deng et al. 2015; Roessner et al. 2010), which display hyperlocomotion, impulsivity, and attention deficits, for investigating the neurobiological basis of ADHD has been proposed and utilized in prior work (Deng et al. 2015). In our current study, we used DAT− mice, which displayed hyperactivity and impaired cognitive flexibility in the OFT and MWM tests. These behavioral alterations exhibited by DAT− mice are consistent with those reported in previous studies (Deng et al. 2015).

The psychostimulants amphetamine (AMPH) and MPH are the most commonly used drugs for treating ADHD (Faraone 2018; Roessner et al. 2010); however, some children do not respond to them adequately or cannot tolerate their adverse effects, such as decreased appetite, increased appetite, difficulty falling asleep, anxiety, dizziness, and nausea (W. X. Zhao et al. 2017). Previous studies have shown that the TCM preparation NDG alleviates some stereotypic behaviors in TS patients and animal models (Li et al. 2009; Li et al. 2020, 2011; Lv et al. 2009; L. Zhao et al. 2020). The TCM preparation NDG was also demonstrated to improve cognitive flexibility impairment in children (Li et al. 2011). The TCM herb *U. rhynchophylla* is the key effective component of NDG, and RHY is an important active tetracyclic oxindole alkaloid isolated from *U. rhynchophylla* (Zhou and Zhou 2010; Yuan et al. 2009); therefore, in the current study, the active ingredient of NDG was utilized to treat ADHD and determine its possible underlying mechanism (He et al. 2014; Song et al. 2012).

The OFT and MWM tests are classical paradigms for studying behavior, specifically the learning ability and memory of experimental animals and the spatial navigation ability of rodents (Lalonde and Strazielle 2009; Amos‐Kroohs, Williams, and Vorhees 2011; Zachariassen et al. 2021). In the present study, we assessed hyperactivity and cognitive flexibility impairment in the experimental mice by the OFT and MWM tests. The DAT− mice exhibited differences in behavior, including a longer distance traveled, a longer amount of time spent moving, and fewer platform crossings, compared with RHY and WT mice in the OFT. These outcomes indicate that DAT− mice display hyperactivity and that RHY treatment alleviated this behavioral change. In the MWM test, DAT− mice exhibited a longer latency to find the platform in the learning phase and spent more time and traveled a greater distance in the target quadrant in the probe test than mice in the RHY and WT groups. These observations indicate that the spatial learning ability and memory of DAT− mice were impaired and that RHY relieved this impairment of cognitive flexibility in the current behavioral experiments.

Neuroinflammation involves the immune system in the CNS and comprises a complex series of immune processes within CNS cells, such as glia and astrocytes. These processes are mediated by cytokines, pattern recognition receptors, and peripheral immune cells and occur in response to harmful stimuli, including pathogens, tissue damage, abnormal stimulation, neurotoxins, infection, or injury (Kerekes, Sanchéz‐Pérez, and Landry 2021; Wilson and Thomas 2022). Neuroinflammation can also cause damage to nervous tissues. The levels of inflammatory CNS mediators can be measured to identify children who may be vulnerable to developing ADHD symptoms. Previous studies have also shown that genetic factors are associated with ADHD and some allergic diseases (Pelsser, Buitelaar, and Savelkoul 2009; Miyazaki et al. 2017). Various theories have been postulated regarding the potential mechanisms underlying ADHD, ADHD comorbidities, and allergic diseases, indicating a relationship among inflammation, immunity, and allergic diseases (Kerekes, Sanchéz‐Pérez, and Landry 2021).

Persistent acute neuroinflammation, chronic neuroinflammation, and related immune responses can also lead to the activation of microglia and astrocytes (Pelsser, Buitelaar, and Savelkoul 2009; Miyazaki et al. 2017; Donfrancesco et al. 2020; Wilson and Thomas 2022), which is associated with ADHD pathology in patients and animal models (Coelho‐Santos et al. 2018; Amini‐Khoei et al. 2019). In recent years, biological research on some neurodevelopmental disorders, such as ADHD and TS, has provided evidence that excessive secretion of proinflammatory cytokines (such as IL‐1β and TNF‐α) results in stereotyped behaviors and hyperactivity (Donfrancesco et al. 2020; Chang et al. 2021; Donfrancesco et al. 2021). As two of the main immune cell types in the CNS, microglia, and astrocytes are activated in response to brain inflammation or injury and trigger the release of toxic cytokines and inflammatory mediators, which contribute to the degeneration of neurons. In this context, inhibiting microglial activation is a key strategy for attenuating inflammatory damage and thus preventing further progression of neurodegenerative diseases. Several studies have demonstrated that RHY can protect neurons against ischemia and glutamate‐ or DA‐induced neuronal damage or death (Coelho‐Santos et al. 2018). RHY has also been exhibited to inhibit LPS‐induced nitrogen monoxide (NO) release in cultured rat primary cortical microglia, possibly through a mechanism associated with regulation of iNOS expression by a series of signaling cascades (Yuan et al. 2009; Song et al. 2012), including the mitogen‐activated protein kinases (MAPKs), extracellular regulated protein kinases (ERK), p38 MAPK, and nuclear factor kappa‐B (NF‐κB) pathways (Song et al. 2012; He et al. 2014). Our prior studies have shown that NDG and the key active component RHY can alleviate hyperactivity in ADHD patients and stereotypic behaviors in TS patients and rat models by decreasing the levels of the inflammatory factors IL‐1 and TNF‐α (Lv et al. 2009; Li et al. 2011; L. Zhao et al. 2020; Li et al. 2020). RHY could also improve cognitive impairment in AD rats (Shao et al. 2015; Yang et al. 2018), and learning and memory impairments by reducing neuroinflammation and inhibiting the activation of the JNK signaling pathway in AD model mice (Xian et al. 2014). In the current work, it was shown that RHY ameliorated hyperactivity and cognitive flexibility impairment and concomitantly decreased the levels of the inflammatory factors TNF‐α and IL‐1β in cortical tissue homogenates from DAT− mice. It was also demonstrated that RHY inhibited the expression of the inflammatory factors TNF‐α, IL‐1β, iNOS, and COX‐2 in microglia and astrocytes induced by LPS in vitro; meanwhile, the correlation analysis also showed a positive relationship between the decreased levels of inflammatory factors and the enhanced behavioral outcomes in OFT and MWM tests after RHY treatment. Hence, NDG and RHY can alleviate hyperactivity and cognitive flexibility impairment and inhibit the expression of inflammatory factors both in vivo and in vitro. Interestingly, RHY inhibited the expression of inflammatory factors of TNF‐α and IL‐1β both in vivo and in vitro but decreased the levels of iNOS and COX‐2 in vitro, which might be different effects of actions in vivo and in vitro.

There were some limitations in this study; however, we only described the positive correlation for RHY between the improvement of cognitive behavior and inhibition of inflammatory factors but did not further prove the specific mechanism. Based on these findings, the mechanisms underlying these outcomes may serve as crucial targets for future research, and there is evidence suggesting that RHY could hold therapeutic value in the treatment of ADHD.

## 5 Conclusion

In conclusion, our study suggests that RHY can alleviate hyperactivity and cognitive flexibility impairment, possibly through inhibition of the inflammatory response. Thus, inhibition of the inflammatory response may be an effective strategy for the clinical treatment of ADHD.

## Author Contributions

**Jijun Li**: investigation, conceptualization, resources project administration, writing–original draft, funding acquisition, software. **Bojun Chen**: methodology, data curation. **Zai‐Wang Li**: methodology, formal analysis, conceptualization. **Yi Wang**: data curation, investigation, methodology. **Ian Alberts**: writing–review and editing, writing–original draft, validation, supervision, visualization. **Kexing Sun**: data curation, investigation. **Xiaohong Li**: conceptualization, methodology, resources, supervision.

## Conflicts of Interest

The authors declare no conflicts of interest.

### Peer Review

The peer review history for this article is available at https://publons.com/publon/10.1002/brb3.70121

## Data Availability

All data may be made available on reasonable request to the corresponding author.
